# Crystallization pathways of liquid-bcc transition for a model iron by fast quenching

**DOI:** 10.1038/srep16956

**Published:** 2015-11-19

**Authors:** Shao-Peng Pan, Shi-Dong Feng, Jun-Wei Qiao, Wei-Min Wang, Jing-Yu Qin

**Affiliations:** 1 College of Materials Science and Engineering, Taiyuan University of Technology, Taiyuan, 030024, China; 2 Shanxi key laboratory of advanced magnesium-based materials, Taiyuan University of Technology, Taiyuan, 030024, China; 3 State Key Laboratory of Metastable Materials Science and Technology, Yanshan University, Qinhuangdao 066004, China; 4 Key Laboratory for Liquid-Solid Structural Evolution and Processing of Materials (Ministry of Education), Shandong University, Jinan 250061, China

## Abstract

We report simulations on the local structural evolution in the liquid-bcc transition of a model iron. Fourteen main Voronoi polyhedra are chosen as the representatives of short-range orders (SROs) and their transformations during crystallization are also investigated. Thus, the crystallization pathways for the main SROs are drawn. Our results also show that the transformations between two SROs in the crystallization pathways can be classified into two categories, first the enlargement of coordination number, second the transformation of local symmetry from five-fold to four-fold. The former reduces the potential energy while the latter increases it. It is found that the potential energy cannot decease monotonously whatever crystallization pathway is chosen to transform the icosahedral SRO to bcc SRO. Therefore, the latter transformation might provide the energy barrier of crystallization. We propose two transformation styles among SROs. All the transformations in the crystallization pathways can be achieved according to the styles. Moreover, the two transformation styles indicates that the bcc structure is more similar to liquid than other crystals. That might be the reason why the first phase nucleated during a rapid cooling process should be bcc crystal.

Crystallization is one of the most important processes in condensed-matter physics and materials science. As liquid is solidified into crystal, evolutions in both symmetry and density take place^1^,^2^,^3^. However, the exact crystallization pathways in the liquid-to-crystal transition remain a focus of debate, with no unified picture so far. Although the classical nucleation theory (CNT) provides a nice framework to understand crystallization, it should be noted that the CNT does not properly describe all the aspects of the nucleation process, especially the detailed structural evolutions. In the CNT description, the initial disordered liquid and final ordered crystal are considered as the only key players of nucleation, and the intermediate structures between liquids and crystals are missing. Previous studies have found several metastable solids to link liquids and final crystals^4^,^5^. In some Lennard-Jones and other soft-potential systems, the body-centered cubic crystal emerges in the early process of crystallization^6^,^7^,^8^,^9^,^10^,^11^ while it is random hexagonal close-packed structure in hard-sphere systems^12^,^13^,^14^,^15^,^16^. Although the crystallization pathways including these metastable solids have been visualized by experiments^17^, there is still a gap between liquids and crystals since the metastable solids are usually crystals. Therefore, questions emerge: how the liquid changes into the metastable solid and why the first phase nucleated during a rapid cooling process should be these metastable solids.

Short-range orders (SRO) in liquids are characterized by dominant five-fold symmetry while in crystal it is four-fold or six-fold symmetry to suit periodical packing^18^. Therefore, besides density variation, symmetry transformation also takes place in the crystallization process. However, do these two variations appear simultaneously or separately? Detailed crystallization pathways from the initial liquid are required. Bond orientational order is usually used to characterize the atomic environment in crystallization^19^,^20^,^21^. It can easily classify atoms into the liquid-like and the solid-like. Voronoi method is anther useful tool to describe the local structure of liquids^22^. Its index (see below) can reflect not only the symmetry but also the density (by the number of nearest neighbors)^23^,^24^. For example, To explain the origin of low bond orientation order peak in pure Fe, P. Ganesh and M. Widom performed a Voronoi analysis of the liquid before and during crystallization^25^. Therefore, to investigate the liquid-crystal transition by Voronoi method might give new insight on the crystallization process.

Considering the problems above, we investigated the evolution of SROs in the liquid-bcc transition of a model iron by performing molecular dynamics (MD) simulations. Voronoi method is used to characterize the local structure. Fourteen main Voronoi polyhedra are chosen as the representatives of SRO and their short-time transformations in the crystallization process are also investigated. Therefore, the crystallization pathways for the main SROs are drawn. Then we explained the pathways according to per-atom energy and atomic movement. We found that bcc structure is more similar to liquid than other crystals from the perspective of atomic movement, which might be the reason why the first phase nucleated during a rapid cooling process should be bcc crystal.

## Results and Discussion

### Crystallization pathways

**Figure 1:**
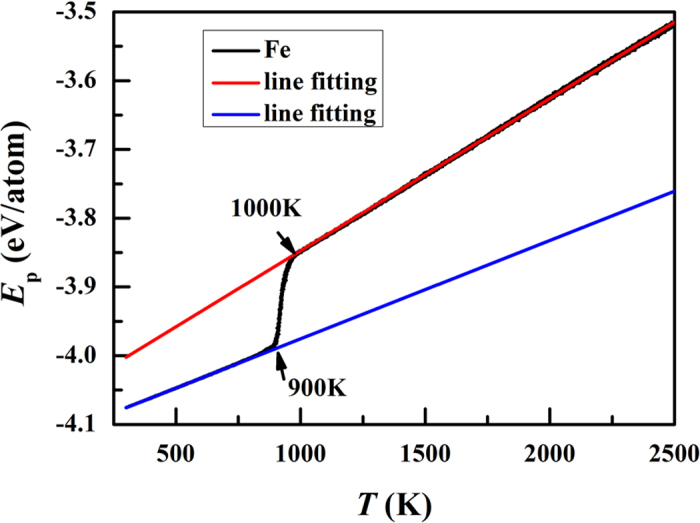
Per-atom potential energy change with temperature in the cooling process.

**Figure 2:**
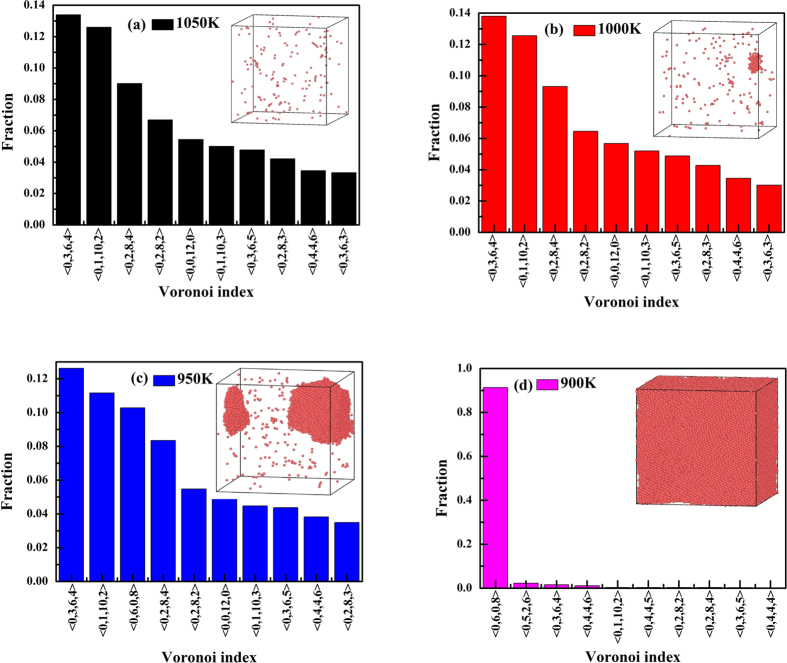
Top ten Voronoi polyhedra for iron at (**a**) 1050 K, (**b**) 1000 K, (**c**) 950 K, and (**d**) 900 K.Inset shows the central atoms of bcc SRO with a Voronoi index of <0,6,0,8>.

Figure 1 shows the per-atom potential energy in the cooling process. It can be seen that the per-atom potential energy changes sharply within the temperature range of 1000 K ~ 900 K, which indicates that the crystallization process occurs in the temperature range. Therefore, we focus on the structural evolution at 1050 K ~ 800 K. Figure 2 shows the top ten Voronoi polyhedra at 1050 K, 1000 K, 950 K, and 900 K, respectively. The Voronoi tessellation method allows determination of the open space around the atom in the polyhedron formed by bisecting the lines joining an atom to its neighboring atoms. A Voronoi polyhedron is described by indices <n_3_, n_4_, n_5_, n_6_> where n_i_ denotes the number of faces with i edges. For example <0,0,12,0> denotes an icosahedron, while <0,0,12,2> denotes a 14-coordinated (Z14) atom, with 12 fivefold bonds and 2 sixfold bonds. The <0,0,12,2> is a characteristic tcp (tetragonal close-packed) structure of the Frank-Kasper type, with a −72° disclination line running through an otherwise perfect icosahedron. In a body-centered cubic crystal all atoms are of Voronoi type <0,6,0,8>, which is an alternate 14-coordinated structure. In the inset of Fig. 2, we show the central atoms of <0,6,0,8>. It can be seen that the distribution of <0,6,0,8> tends to be random at 1050K while a crystalline nuclear appears at 1000 K. At 950 K, the crystal grows large and the crystallization process is close to the end at 900 K. The icosahedral SRO has a Voronoi index of <0,0,12,0>, which is well-known to play an important role in stability of liquid structure^27^. The polyhedra with the index of <0,1,10,2> and <0,2,8,2> can be regarded as deformed-icosahedral SROs. These SROs play an important role in the stability of liquid structure. Other SROs might be intermediate states from icosahedral to bcc SROs.

**Figure 3:**
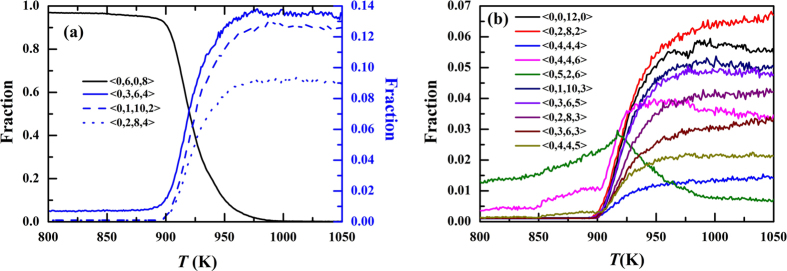
The temperature dependence of main polyhedra. The populations of <0,5,2,6> and <0,4,4,6> increase at the beginning of the crystallization process while other poyhedra except <0,6,0,8> decrease, indicating the important role of <0,5,2,6> and <0,4,4,6> in the crystallization process.

Figure 3 displays the temperature dependence of the populations of the fourteen Voronoi polyhedra shown in Fig. 2. In Fig. 3(a), the fraction of <0,6,0,8> rapidly increase from nearly 0.0 to more than 0.9, especially in the range of 1000 K ~ 900 K, indicating the main crystallization process. The populations of the top three SROs at 1050 K, <0,3,6,4>, <0,1,10,2> and <0,2,8,4>, decrease in the crystallization process. However, unlike <0,1,10,2> and <0,2,8,4>, the fraction of <0,3,6,4> does not reach to nearly zero when the crystallization process comes to the end. This feature suggests that the polyhedron of <0,3,6,4> might play an important role in the crystallization process. Figure 3(b) shows the variation of other ten main Voronoi polyhedra during the crystallization process. The number of <0,5,2,6> in the range of 1050 K ~ 917 K and <0,4,4,6> in the range of 1050 K ~ 950 K increase as the temperature decreases. Moreover, when the crystallization process is close to the end, the fractions of these two polyhedra are still rather large. These facts indicate these two kinds of polyhedra may play an important role to connect the structures of liquid and bcc crystal. Fractions of icosahedral or deformed-icosahedral polyhedra, <0,0,12,0>, <0,1,10,2> and <0,2,8,2> rapidly decrease to nearly 0.0 at 900 K. The populations of other polyhedra also decrease and reach to nearly zero. These facts indicate that these polyhedra might be located at the forepart of the crystallization pathways.

**Figure 4:**
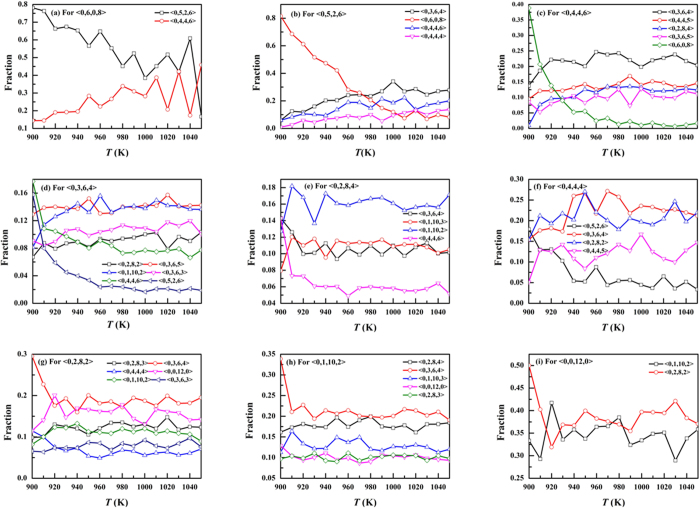
The transformations of main polyhedra. At each temperature, we compared the initial configuration with the one equilibrated after 10 fs and got the transformed polyhedra. For each transformed polyhedron, we investigated the fractions of objective polyhedra.

To determine the crystallization pathways, we investigated the transformations of the fourteen polyhedra during the crystallization process. At each temperature, we compared the configuration and the one after 10 fs, and the fractions of objective polyhedra for nine transformed polyhedra were shown in Fig. 4. It is known that, when the crystallization process occurs, the melting process also occurs. Firstly we studied the transformation of <0,6,0,8> in Fig. 4(a). There are two main objective polyhedra, <0,5,2,6> and <0,4,4,6>, for the transformed <0,6,0,8>. It is known that, when the crystallization process occurs, the melting process also occurs. Then we investigated the transformations of <0,5,2,6> and <0,4,4,6> in Fig. 4(b,c), respectively. It can be seen that, as the temperature decreases, the fractions of objective polyhedron <0,6,0,8> for both of the transformed polyhedra increase and become the top one at the end, confirming the final part of the crystallization pathways. The fraction of objective polyhedron <0,6,0,8> for <0,5,2,6> is much larger than that for <0,4,4,6>. Therefore, the polyhedron of <0,5,2,6> is more easily transformed into <0,6,0,8> than <0,4,4,6>. Besides <0,6,0,8>, <0,5,2,6> is also transformed into <0,4,4,4> and <0,3,6,4>, and <0,4,4,6> is also transformed into <0,3,6,4>, <0,2,8,4>, <0,4,4,5> and <3,6,5>. Then we referred to the transformation of <0,3,6,4>, <0,4,4,4> and <0,2,8,4> in Fig. 4(d–f). It can be seen that, besides <0,4,4,6> and <0,5,2,6>, many of them are transformed into <0,1,10,2> and <0,2,8,2>. Finally, we investigated the transformations of <0,1,10,2>, <0,2,8,2> and <0,0,12,0> in Fig. 4(g–i). It can be found that the icosahedral SRO is usually transformed into <0,1,10,2> and <0,2,8,2>. We also study the transformations of <0,1,10,3>, <0,2,8,3>, <0,3,6,3>, <0,4,4,5> and <0,3,6,5> (not shown).

**Figure 5:**
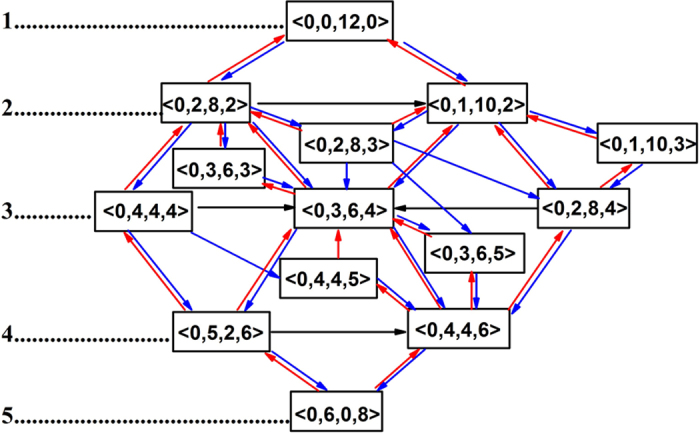
Crystallization pathways from an icosahedral SRO to a bcc one. The blue arrow stands for the crystallization process while the red arrow corresponds to the melting process. The numbers in the left is the group ID. The black arrow represents the transformations within the same group.

**Figure 6:**
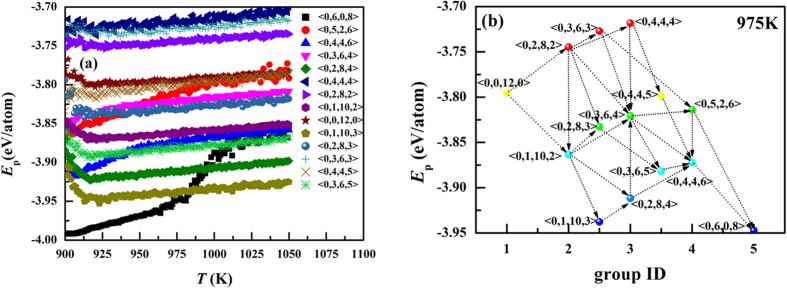
(**a**) Per-atom potential energy for the central atoms of fourteen polyhedra as a function of temperature. (**b**) The crystallization pathways with per-atom potential energy at 975 K.

According to the transformations of the fourteen polyhedra, a crystallization pathway was drawn in Fig. 5. The nine polyhedra in Fig. 4 were classified into five groups. The Voronoi polyhedra in two adjust groups can be transformed into each other. The plyhedra of <0,1,10,3>, <0,2,8,3>, and <0,3,6,3> play an important role to connect group 2 and 3 while the polyhedra of <0,4,4,5>,and <0,3,6,5> take a bridge between group 3 and 4. Therefore, we classified <0,1,10,3>, <0,2,8,3>, and <0,3,6,3> into group 2.5, and <0,4,4,5>and <0,3,6,5> into group 3.5. The blue arrow stands for the crystallization process while the red arrow corresponds to the melting process. Figure 4(i) shows that the icosahedral SRO is mainly transformed into <0,1,10,2> and <0,2,8,2>. Figure 4(h–g) display that <0,1,10,2> and <0,2,8,2> both take <0,3,6,4> as the first choice to be transformed into. However, for the polyhedra in group 3, the polyhedra in group 4 are not the preferable objective polyhedra. They tend to be transformed into the polyhedra in group 2 or also in group 3. For the polyhedra in group 4, the polyhedra in group 3 prefer to be transformed into <0,4,4,6>. However, the polyhedron of <0,5,2,6> is more easily transformed into <0,6,0,8> than <0,4,4,6>. These facts indicate that the transformation from group 3 to group 4 plays a key role in the crystallization process and might be the main block in the liquid-bcc transition. Per-atom potential energy. To study the reason for the crystallization pathways, we investigated the per-atom potential for the central atoms of fourteen polyhedra in the temperature range of 1050 K-900 K in Fig. 6(a). It can be seen that the per-atom potential for the central atoms of bcc SRO, <0,6,0,8>, is not the lowest one at the beginning of crystallization. As the temperature decreases and the crystal is growing, it becomes the lowest one. The icosahedral SRO is considered to play an important role in the stability of liquid structure. However, its per-atom potential energy is rather high. In fact, for liquid iron, the per-atom potential energy decreases as the coordination number increase, *E*_p_(CN14) < *E*_p_ (CN13) < *E*_p_(CN12). Moreover, for a certain CN, the per-atom potential energy increases with the decreasing local five-fold symmetry which can be measured by *d*_5_ = *n*_5_/CN. The pathways from icosahedral to bcc SRO can be classified into two categories: (1) enlargement of CN from 12 to 14. (2) transformation of local symmetry from five-fold to four-fold. The former might reduce potential energy while the latter might increase potential energy. Figure 6(b) shows the crystallization pathways with per-atom potential energy at 975 K. It can be seen that, whatever crystallization pathway is chosen to transform the icosahedral SRO to bcc SRO, the potential energy cannot decrease monotonously. Therefore, the symmetry transformation might provide the energy barrier between liquid and bcc crystal.

### Atomic movement

**Figure 7:**
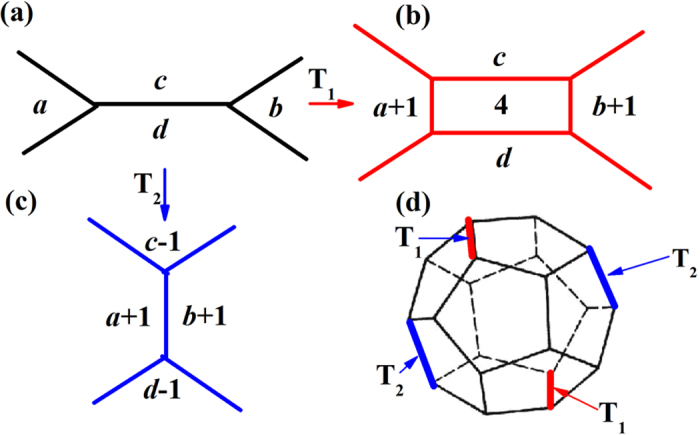
(**a,b**) is the first transforming form (T_1_), and (**a,c**) is the second one (T_2_). The numbers in the brackets in (**a–c**) are the number of edges of the faces. (**d**) the positions where T_1_ and T_2_ might happen for <0,0,12,0> transformed to <0,6,0,8>.

Although the main crystallization pathways and the potential energy change are studied, the detail of transforming process is still a secret. Figure 7 shows two possible transformation styles between two polyhedra^28^,^29^. Figure 7(a),7(b) shows that an extra atom approaches the central atom of a polyhedron from an edge of the polyhedron and creates *SRO* a very small quadrilateral face at the edge. Therefore, the coordination number for the central atom increases. We call this transformation style T_1_. Another transformation style T_2_ is shown in Fig. 7(a,c). In this transformation style, no extra atom is introduced. Here, we use the four numbers together with another number *t* to form an index (*t*, *a*, *b*, *c*, *d*) to describe the transformations. The numbers in the brackets, *a*, *b*, *c*, and *d*, are the number of edges of the faces. *t* = 1 represents T_1_ and *t* = 2 corresponds to T_2_. *T* = −1 is the inverse process of T_1_. As the number of edges in the face of a Voronoi polyhedron is usually 4, 5 or 6, we labeled all the possible transformations based on the two transformation styles as follows:


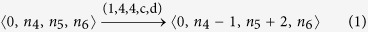



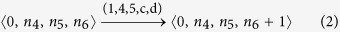



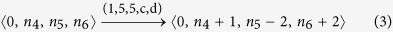



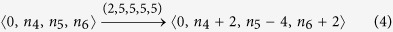



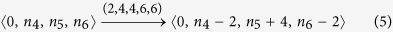



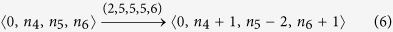



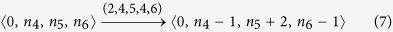



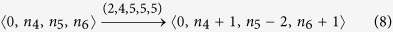



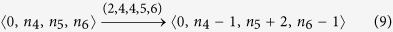


**Figure 8:**
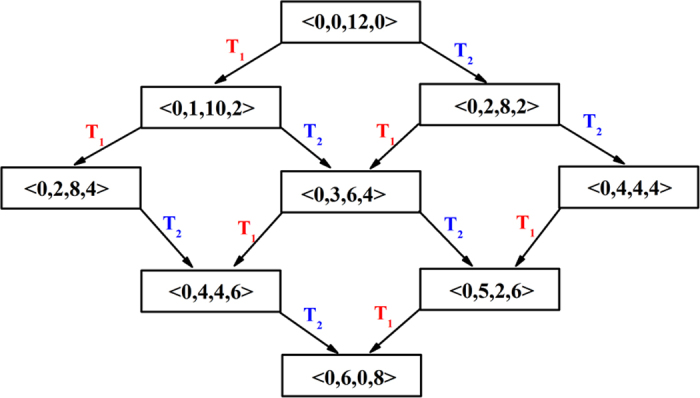
The crystallization pathways from an icosahedral cluster to a bcc cluster by T_1_ and T_2_. The numbers, (**a–d**) in all the T_1_ and T_2_ equal to 5.

Transformation 1 to 3 belong to T_1_ while the others correspond to T_2_. Transformation 4 and 5, 6 and 7, and 8 and 9 are inverse to each other, respectively. Transformation 6 and 8, and 7 and 9 have the same effect. Based on the transformation styles, we proposed a model for the transformation pathways from an icosahedral to a bcc cluster. Figure 7(d) shows the Voronoi polyhedron with icosahedral packing. We selected four edges as the possible positions where the transformation might happen for <0,0,12,0>. Two T_1_ and Two T_2_ can change <0,0,12,0> into <0,6,0,8>. The possible transformation pathways might be described in Fig. 8. It can be seen that all the nine polyhedra in Fig. 4 are involved in the crystallization pathways. In fact, the transformation among the polyhedra in Fig. 5 can all be described by transformation 1 to 9. Therefore, the crystallization pathways in Fig. 5 can be achieved by the two transformation styles. Comparing elemental Fe to Cu^25^,^30^, Ganesh and Widom find that the degree of icosahedral order is greater in Fe than in Cu, possibly because icosahedral disclination line defects are more easily incorporated into bcc environments than fcc. Therefore the current study is consistent with the results by Ganesh *et al*.

Most SROs in liquid with the Voronoi index of <*n*_3_,*n*_4_,*n*_5_,*n*_6_> have several characteristics: *n*_3_ = 0; *n*_4_*2+*n*_5_ = 12; *n*_5_ is the largest one^31^. All the SRO satisfying the conditions above can be transformed into each other by different number of T_1_ and T_2_. It can be seen that the bcc SRO with the index of <0,6,0,8> satisfies the first two conditions of SRO in liquids while the fcc or hcp SRO with the index of <0,12,0,0> only satisfies the first one. This feature indicates that the bcc structure is more similar to liquid than fcc and hcp structures. Therefore, it is easier for liquid structure to be transformed into bcc structure. Maybe it is the reason for Alexander and McTague’s proposition that the first phase nucleated during a rapid cooling process should be bcc crystallite^32^ from the point of structure.

## Conclusion

In summary, we performed MD simulations to investigate the evolution of SRO in the process of liquid-bcc transition for pure iron. Fourteen main polyhedra were studied in the crystallization process. Their populations and transformations as a function of temperature were studied and a crystallization pathway was drawn. It is found that <0,5,2,6> and <0,4,4,6> play an important role in the final part of the crystallization pathways. However, they are not the first choice to be transformed into for other polyhedra, which might be the block in the crystallization process. We also investigate the per-atom potential energy of the central atoms of the fourteen polyhedra. Enlargement of CN might reduce potential energy while transformation of local symmetry from five-fold to four-fold might increase potential energy. Therefore, the latter might be the energy barrier for crystallization. Finally, The possible atomic movement for the transformation from an icosahedral cluster to a bcc cluster was discussed. Two transforming styles were verified and all the fourteen clusters could be transformed into bcc clusters after different steps of these two styles.

## Methods

The MD simulation was carried out by using large-scale atomic/molecular massively parallel simulator (LAMMPS)^33^ based on embedded atom method (EAM) potential^34^. The simulation was performed for a cubic box with 100,000 Fe atoms and subjected to periodic boundary condition. The *NPT* canonical ensemble was applied and the time step is 1 fs. The initial configuration was a random one and the system was equilibrated for 1 ns at 2500 K and then quenched to 300 K with the cooling rate of 10^12^ K/s. Voronoi polyhedron analysis^26^ was performed to describe SRO in the crystallization process. The Voronoi polyhedron index is expressed as <*n*_3_, *n*_4_, *n*_5_, *n*_6_>, where *n*_*i*_ denotes the number of *i*-edged faces of the Voronoi polyhedron and also represents *i*-fold symmetry structures. The summation of *n*_*i*_ corresponds to the coordination number (CN) of the central atom.

## Additional Information

**How to cite this article**: Pan, S.-P. *et al*. Crystallization pathways of liquid-bcc transition for a model iron by fast quenching. *Sci. Rep*. **5**, 16956; doi: 10.1038/srep16956 (2015).
